# The Role of Long Intergenic Noncoding RNA in Fetal Development

**DOI:** 10.3390/ijms252111453

**Published:** 2024-10-25

**Authors:** Ifetoluwani Oluwadunsin Oguntoyinbo, Ravi Goyal

**Affiliations:** 1 School of Animal and Comparative Biomedical Sciences, College of Agriculture, Life & Environmental Sciences, University of Arizona, Tucson, AZ 85721, USA; ioguntoyinbo@arizona.edu; 2 Department of Obstetrics and Gynecology, College of Medicine, University of Arizona, Tucson, AZ 85724, USA

**Keywords:** DOHaD, fetal origins, neonatal development, fetal programming, epigenetic

## Abstract

The role of long intergenic noncoding RNAs (lincRNAs) in fetal development has emerged as a significant area of study, challenging the traditional protein-centric view of gene expression. While messenger RNAs (mRNAs) have long been recognized for their role in encoding proteins, recent advances have illuminated the critical functions of lincRNAs in various biological processes. Initially identified through high-throughput sequencing technologies, lincRNAs are transcribed from intergenic regions between protein-coding genes and exhibit unique regulatory functions. Unlike mRNAs, lincRNAs are involved in complex interactions with chromatin and chromatin-modifying complexes, influencing gene expression and chromatin structure. LincRNAs are pivotal in regulating tissue-specific development and embryogenesis. For example, they are crucial for proper cardiac, neural, and reproductive system development, with specific lincRNAs being associated with organogenesis and differentiation processes. Their roles in embryonic development include regulating transcription factors and modulating chromatin states, which are essential for maintaining developmental programs and cellular identity. Studies using RNA sequencing and genetic knockout models have highlighted the importance of lincRNAs in processes such as cell differentiation, tissue patterning, and organ development. Despite their functional significance, the comprehensive annotation and understanding of lincRNAs remain limited. Ongoing research aims to elucidate their mechanisms of action and potential applications in disease diagnostics and therapeutics. This review summarizes current knowledge on the functional roles of lincRNAs in fetal development, emphasizing their contributions to tissue-specific gene regulation and developmental processes.

## 1. Introduction

The fundamental tenet of gene expression explains that messenger RNA (mRNA), transcribed from DNA, is translated into proteins that perform structural, regulatory, and functional roles in various cellular processes [1]. Jacob and Monod’s 1961 study on genetic regulatory mechanisms established the crucial role of mRNAs in transmitting genetic information [2]. However, the protein-centric theory was challenged when it was discovered that protein-coding genes in both lower and higher organisms are similar, yet this similarity could not account for the complexity observed in higher organisms [3].

The 2004 International Human Genome Sequencing Consortium revealed that the human genome contains only about 20,000 protein-coding genes, constituting just over 2% of the total genomic sequences. Comprehensive annotation endeavors, such as the ENCODE (Encyclopedia of DNA Elements, 2012) initiative, have expanded our understanding of the hidden facets of the genome. Additionally, the FANTOM (Functional Annotation of Mouse Genome) project discovered approximately 35,000 non-coding transcripts from about 10,000 different loci [4,5,6].

Over the past decade, several short non-coding RNAs have been thoroughly classified, including microRNA, small interfering RNA (siRNA), small nuclear RNA (snRNA), small nucleolar RNA (snoRNA), and Piwi-interacting RNA (piRNAs). However, long non-coding RNAs (lncRNAs) have remained relatively unexplored due to their varying lengths, from 200 nucleotides to over 100 kilobases, and their comparatively low cellular abundance [7,8]. With the advent of high-throughput technologies, it has been shown that most of the human genome is transcribed, surpassing the present gene designations.

The revolutionary advancements in next-generation sequencing (NGS) and its applications, such as RNA-seq, have addressed some of these challenges by capturing whole transcriptome data, including lncRNAs [8]. These discoveries have significantly enhanced our understanding of the functional properties of what was previously assumed to be junk DNA, revealing the crucial roles of non-coding RNAs (ncRNAs) [9,10].

## 2. What are LncRNAs?

LncRNAs are non-coding transcripts that are more than 200 bp and are similar in structure to protein-coding genes, and these transcripts have many mRNA signatures. LncRNAs, like mRNA, are transcribed by RNA polymerase II and can undergo the processes of splicing, polyadenylation, and 5′ capping [11]. Like protein-coding genes, lncRNAs possess histone marks of active promoters and actively transcribed gene bodies, but they have little to no open reading frames (ORFs). Importantly, the nucleus contains the bulk of lncRNAs [12]. However, in contrast to mRNA, lncRNAs can fold into complex secondary and tertiary structures, which allows them to act as sponges for miRNA or serve as a scaffold that enables protein regulatory complexes [13]. Interestingly, it has been documented that lncRNAs control several cellular functions, including differentiation, proliferation, and apoptosis. They perform several biological functions that involve modulating proteins or multiprotein complexes, binding DNA/RNA-associated proteins to regulate transcriptional expression, regulating DNA stability through the formation of r-loop and triple helices, interacting with chromatin complexes to help regulate epigenetic gene expression, and ultimately helping to form a higher-order chromatin structure [14].

## 3. Classification of LncRNAs

The human genome comprises non-coding regions interspersed among coding sequences, which are classified into several subtypes [13,15,16]. LncRNAs are transcribed from distinct genomic loci and may exhibit various poly(A) modifications, resulting in localization to different cellular regions [17]. LncRNA is classified based on genomic location and the context of protein-coding genes. LncRNA transcribed from the intergenic regions (the regions between two protein-coding genes) is called long intergenic non-coding RNA (lincRNA) [18]. LncRNA transcribed from the introns of protein-coding genes is called intronic lncRNA. Similarly, sense lncRNAs are transcribed from the sense strand of protein-coding genes and may contain exons that overlap partially or completely with the coding gene’s sequence. In contrast, antisense lncRNAs are transcribed from the antisense strand of protein-coding genes [19].

Research indicates that lncRNAs are predominantly localized in the nucleus and associated with chromatin, suggesting they may significantly impact DNA sequences [19]. LncRNAs regulate chromatin structure in three functional phases: chromatin remodeling, DNA methylation, and histone modifications. Despite lacking basic sequence conservation, lncRNAs perform these functions across a broad evolutionary spectrum [13,20]. A study found that approximately 42% of the 182 assessed lncRNAs are involved in transcriptional regulation [18]. Furthermore, lncRNAs can be classified into trans-lncRNAs, which regulate the expression of distant genes, and cis-lncRNAs, which affect the expression of genes in close genomic proximity [18].

## 4. LincRNAs

This review focuses on lincRNAs, which are transcribed entirely from regions between two protein-coding genes. LincRNAs typically possess their own promoters and are well conserved. Most lincRNAs are transcribed by RNA polymerase II and undergo post-transcriptional modifications, including 5′ capping and 3′ polyadenylation [21]. They also exhibit ribosome occupancy akin to the 5′UTRs of protein-coding genes and share similar promoter and transcriptional features [13,22].

The existence of lincRNAs was first suggested by novel sequences identified using tiling arrays, which revealed widespread transcriptions from non-coding regions that do not overlap with protein-coding genes [5,16,23,24,25]. Since they are transcribed from distinct loci, their expression profiles and the effects of their overexpression or down-regulation are relatively straightforward to interpret [26,27].

Numerous human lincRNAs have been discovered in genomic regions outside the well-characterized protein-coding regions. These lincRNAs exhibit intriguing properties, including associations with various diseases, tissue-specific expressions, and changes during their development. Understanding the role of lincRNAs in regulating complex biological processes is an exciting area of research [8]. According to Lncipedia, a comprehensive lncRNA database, high-throughput transcriptome studies have cataloged over 111,000 lncRNA transcripts, with approximately 50% being lincRNAs originating from intergenic regions [28].

## 5. Conservation of LincRNAs

A notable feature of lincRNAs is their high level of conservation across mammals [29,30,31]. While many lncRNAs are regarded as transcriptional noise due to their poor conservation, numerous lincRNAs are classified as ultra-conserved or highly conserved elements in mammals. The high level of conservation suggests that the removal of these ultra-conserved elements in vivo could have substantial phenotypic effects due to functional constraints [32,33]. However, a previous study found that deleting four different ultra-conserved elements in mice did not affect their viability or produce abnormal phenotypes [34]. Further research is needed to fully understand the roles of this highly conserved group of lincRNAs.

The similarity between mouse and human lincRNAs can vary depending on the annotation method used. Approximately half of the non-coding transcripts from mouse cDNA libraries map to the human genome [35]. Surprisingly, only about 14% show evidence of expressed sequence tags or cDNA indicating a human orthologous transcript [11]. This discrepancy raises questions about whether lincRNA functions are truly conserved and has led to the development of alternative annotation methods [21,36].

Using ChIP-seq signatures of histone H3 lysine 4 tri-methylation (H3K4me3) and histone H3 lysine 36 tri-methylation (H3K36me3), known as “K4-K36” clusters, Guttman et al. identified approximately 1700 transcriptional units greater than 5 kb in four mouse cell lines. These findings were validated using tiling microarrays, PCR, and northern blots. LincRNAs can regulate the expression of their target genes or modulate the epigenetic landscape of chromatin. Transcriptionally active lincRNAs are characterized by the presence of the “K4-K36” domain, with histone H3K4 trimethylation at their 5′ end and histone H3K36 trimethylation in the gene body, which indicates active transcription [29,30,31,37]. Notably, about 70% of lincRNAs with the “K4-K36” domain exhibit RNA transcription activity, a percentage comparable to that of protein-coding genes (~72%). The most significant finding is that the orthologous regions in mice retain approximately 70% of the transcriptionally active domains (K4-K36 domain) found in human lincRNAs, which is similar to the proportion of protein-coding genes (80%) [29]. This chromatin signature was later applied to human cell lines, revealing that, alongside HOTAIR, 20% of lincRNAs are associated with Polycomb repressive complex 2 (PRC2) [29].

## 6. Tissue Specificity and Developmental Patterning

Across a broad spectrum of organisms, lincRNAs play crucial functional roles. The differential expression of lincRNAs has been observed in various tissues, diseases, and cellular responses, highlighting their significant tissue specificity and potential to fine-tune target gene expression in a tissue-specific manner [38]. Additionally, lincRNAs exhibit disease-specific expression patterns [39]. In *Saccharomyces cerevisiae* (budding yeast), lincRNAs are involved in stress responses, including nutrient starvation [40]. In *Arabidopsis thaliana*, while lincRNAs are expressed at lower levels compared to protein-coding genes, some subsets display tissue-specific and stress-responsive expression patterns, suggesting their roles in metabolic and tissue-specific processes across invertebrate species [41,42]. These characteristics of lincRNAs have prompted efforts to utilize them in translational and clinical applications, such as disease biomarkers [8].

Recent RNA-sequencing (RNA-seq) data from 30 tissues, provided by the Genotype-Tissue Expression (GTEx) consortium, revealed that the median tau score for the tissue specificity of lincRNAs is 0.90, which is significantly higher than 0.77 for mRNAs [29]. Importantly, unsupervised clustering analyses showed that 78% of lincRNAs are tissue-specific, in contrast to only 19% of mRNAs. This trend remains consistent regardless of expression levels, with higher specificity scores seen in more highly expressed lincRNAs. This strong tissue specificity implies that lincRNA expression is tightly regulated, supporting the idea that lincRNAs play a crucial role in defining cell state [29]. Pioneering studies of lincRNA expression in mouse brains have also unveiled their precise patterns of expression in specific tissues, cell types, and subcellular compartments [43]. Mouse lincRNA knockout models have identified lincRNAs associated with perinatal or postnatal lethality, as well as growth and organ-specific developmental defects, which may not be directly translatable to humans [44]. However, assigning definitive functions to lincRNAs based on knockout phenotypes is challenging, as observed phenotypes may result from the disruption of gene regulatory elements within lincRNA loci. While murine genetic studies offer valuable insights into human lincRNA biology, they are constrained by evolutionary differences and the complexities of excluding RNA-independent effects [35]. Existing evidence suggests that lincRNAs play crucial roles in diverse biological processes, particularly in regulating gene expression during embryonic development. Many lincRNAs are specifically expressed in embryonic stem cells, neural progenitor cells, and tissues during development, indicating their potential involvement in differentiation and lineage specification [45,46]. Additionally, lincRNAs have been implicated in the regulation of the Hox gene clusters, which are essential for patterning the anterior-posterior axis during embryogenesis. For instance, the lincRNA HOTAIR is involved in the epigenetic silencing of the HOXD locus, thereby contributing to the correct patterning of the body plan [46]. Furthermore, the lincRNA Xist is required for the initiation and maintenance of X chromosome inactivation, a critical process for dosage compensation in female mammals [47].

In summary, the discovery of a vast transcriptional landscape in the genome primarily comprises lincRNAs, which are involved in various biological processes [1,21,48,49]. These recent findings have significantly expanded our understanding of gene regulation. LincRNAs have emerged as a crucial regulatory layer, playing important roles in diverse biological processes, including fetal development.

## 7. Roles in Fetal Development

The advent of next-generation RNA sequencing technologies has enabled high-throughput analyses of lincRNA expression across different cell types and tissues [50,51]. These studies have shown that lincRNAs often cluster near transcription factors and developmental genes [52,53,54]. Despite these insights, many mouse models involving lincRNAs have exhibited only minor defects or no notable phenotypic features [55,56,57,58,59]. Early studies on the knockout strains of Xist and Tsix demonstrated the essential role of lincRNAs in X inactivation and viability. Subsequent research in the 2000s identified HOTAIR as a key regulator that represses the transcription of HOX family genes [60]. These pioneering studies spurred interest in exploring lincRNA functions within specific cellular contexts, developmental stages, and diseases [61].

## 8. Role of LincRNA in Embryonic Development

Yan et al. identified the expression of 3405 lncRNAs, including 2733 likely lincRNAs, through an RNA-seq analysis of single cells from human pre-implantation embryos and embryonic stem cells [62]. While several of these lincRNAs exhibited distinct expression patterns based on developmental stages, the specific profiles of lincRNAs during embryonic development were not analyzed in detail. However, they did provide developmental stage-specific RNA-seq data for human pre-implantation embryos [63,64].

In bovine embryos, the knockdown of specific lincRNAs affected embryo growth, leading to increased developmental rates and larger blastocysts [65]. Early-stage spliced lincRNAs were also predominantly indicated. Conversely, in mice, the siRNA-mediated silencing of a promoter-associated lincRNA, specifically expressed in two-cell stage embryos, resulted in embryonic lethality. This phenotype was rescued by overexpressing the interacting protein [66].

Large-scale RNA sequencing and genomics studies, combined with a comprehensive candidate selection pipeline, have successfully identified physiologically essential lincRNAs. The initial characterization of 18 lincRNA knockout strains in mice revealed perinatal or postnatal lethal phenotypes, as well as growth and organ-restricted developmental defects not easily replicated in humans. For instance, the Fendrr, Peril, and Mdgt mutant strains displayed perinatal or postnatal lethality, with Fendrr mutants showing defects in the lungs, heart, and gastrointestinal tract. Notably, lincRNAs such as Fendrr, transcribed divergently from the transcription factor Foxf1, are crucial for organ development during embryogenesis [44]. Additional mutants like linc–Brn1b and linc–Pint exhibited growth defects, with linc–Brn1b−/− mutants showing abnormalities in upper layer II-IV neuron generation in the neocortex. These findings underscore the critical roles of lincRNAs in fetal development and set the stage for future large-scale functional investigations [44].

In a survey of 20 knockout mouse lines [67], mice homozygous for the Fendrr gene deletion survived birth but died shortly thereafter due to severe respiratory issues. At the embryonic stage E13.5, the developing lungs of these knockout embryos were notably small, with disorganized, globular lobes. The perinatal lethal phenotype of Fendrr mutants was consistent across two different genetic backgrounds: a C57BL6/129 hybrid and mice further backcrossed to C57BL/6,. The study also identified a range of phenotypes, from perinatal lethality to defects resembling premature aging, including abnormalities in the lungs, skeleton, and muscle [67].

## 9. Fetal Tissue-Specific Development

In addition to their roles in early embryonic stages, lincRNAs are crucial for tissue-specific development during fetal stages.

## 10. Role in Fetal Nervous System Development

Research leveraging RNA sequencing and genomic data has uncovered the previously unknown roles of lincRNAs in brain development. Studies demonstrate that the regulation of brain-specific lincRNAs significantly impacts the differentiation capacity of neural progenitors. Key lincRNAs involved in neural development include Peril, Evf2 (Dlx6os1), and linc-Brn1b [44,68]. For instance, Evf2 acts as a transcriptional coactivator through Dlx2, enhancing the transcriptional activity of Dlx5/6 and glutamate decarboxylase 1 (Gad1), which is essential for converting glutamate to GABA. This regulation is crucial for the development of GABAergic interneurons in the mouse brain [69]. Silencing Evf2 results in abnormal synaptic activity due to the disrupted formation of GABAergic circuits in the hippocampus and dentate gyrus [68].

Notably, the homeodomain transcription factor Emx2, expressed in the dorsal telencephalic neuroepithelium (the region giving rise to cortical neurons), is surrounded by clusters of lincRNAs, highlighting the need for further exploration in brain tissue [70,71]. The complex regulatory mechanisms of lincRNAs likely evolved to facilitate the layered control of gene expression essential for the brain’s cellular diversity and intricate functions. For example, Linc–Brn1b is a lincRNA that influences cerebral cortex development. Many other lincRNAs, besides Linc–Brn1b, exhibit restricted patterns of expression within progenitors in the telencephalon’s ventricular zone (VZ) and subventricular zone (SVZ), suggesting their roles in neurogenesis. Additionally, some lincRNAs display dynamic and cell-specific expression patterns across different brain regions [43].

## 11. Role in Fetal Cardiovascular Development

LincRNAs are also pivotal in regulating cardiac chromatin structure during heart development. They are involved in governing cardiac development, orchestrating transcriptional programs, and responding to stress, presenting potential biomarkers and therapeutic targets for cardiovascular diseases [72,73,74].

One prominent example is the mouse-specific lincRNA Braveheart (Bvht). Bvht controls the differentiation of mesodermal precursors into cardiomyocytes by regulating the expression of the mesoderm posterior basic helix–loop–helix transcription factor 1 (MesP1). It does so through interaction with the PRC2 histone modifier, sequestering the polycomb protein Suz12 from the promoters of cardiac lineage-determining genes such as Hand1, Hand2, Mesp1, Nkx2-5, and Tbx20 [73]. Bvht’s function is crucial for cardiomyocyte development, as its depletion disrupts the expression of cardiac-specific genes and impairs cardiomyocyte maturation [75].

Despite these findings, the comprehensive annotation of lincRNAs is still limited, and only a few have been studied in the context of heart development and cardiovascular diseases [76]. To advance our understanding of cardiac lincRNAs, which are expressed during heart development, genome-wide identification and characterization are essential. Wang et al. conducted a genome-wide RNA sequencing profiling of zebrafish embryonic hearts, adult hearts, and adult muscle, generating a high-confidence set of 813 cardiac lincRNA transcripts, with 423 being novel. Of these, 564 are expressed in the embryonic heart and 730 in the adult heart, including two novel lincRNAs, TCONS_00000891 and TCONS_00028686, which exhibit cardiac-enriched expression patterns. They also identified 51 lincRNAs with differential expressions between embryonic and adult hearts, including TCONS_00009015, which responds to doxorubicin-induced cardiac stress. This systematic identification and characterization of cardiac lincRNAs provides a foundation for future studies on their roles in heart development and cardiovascular diseases [77].

## 12. The Roles of LincRNAs in Fetal Reproductive System Development

### 12.1. Spermatogenesis and Testicular Development

LincRNAs play crucial roles in testicular function and spermatogenesis. Microarray studies have identified numerous lincRNAs in neonatal and adult mouse testes, many of which overlap or are located near genes coding for transcription factors involved in spermatogenesis. These lincRNAs exhibit high expression levels in testicular and neural tissues, suggesting a significant role in these tissues despite their generally low transcription levels. The regulation of lincRNA expression implies their importance in testicular function, though many of their functions remain to be elucidated [78,79,80]. A systematic expression profiling study of lincRNAs during post-natal development of the mammalian testis has used genome-wide techniques to explore these roles further [78].

### 12.2. Ovarian Development

Systematic analyses of lincRNAs in ovarian tissues are limited, but emerging technologies such as stranded sequencing and the development of PCR and hybridization arrays are likely to increase our understanding. One specific ovarian lincRNA, NORFA, has been implicated in granulosa cell apoptosis, follicular atresia, and sow fertility [81,82]. While there is evidence that lincRNAs are involved in regulating female reproduction, the full extent of their involvement in ovarian functions and fertility remains incomplete.

## 13. The Role of LincRNAs in Fetal Pancreas Development

LincRNAs play significant roles in pancreas organogenesis and islet cell development. During early pancreas development, lincRNAs are involved in regulating the fate of pancreatic progenitor cells. Specifically, the lincRNA H19 has been shown to interact with chromatin-modifying complexes to regulate the expression of developmental genes. H19 is known to affect the expression of genes involved in cell proliferation and differentiation by modulating chromatin accessibility [83,84].

The differentiation of endocrine cells, such as insulin-producing β-cells, is a crucial step in pancreas development. Several lincRNAs are involved in this process. The lincRNA mouse βlinc1, the orthologue of HI-LNC15, has been identified as a key regulator of β-cell differentiation. βlinc1 modulates the expression of the transcription factor NKX2.2, which is essential for β-cell development. By influencing NKX2.2 levels, βlinc1 helps balance progenitor cell proliferation and differentiation into mature β-cells [85]. The knockout of βlinc1 in mice resulted in glucose intolerance, a lower number of insulin-positive β-cells, and an increased number of somatostatin-positive δ-cells. This indicates βlinc1’s role in the correct specification of endocrine precursor cells during pancreas development, particularly in β-cell differentiation [85].

Recent studies have also described βlinc2 and βlinc3. βlinc2 is upregulated in response to high-fat diets and in db/db mice, correlating with increased weight and glycemia, while βlinc3 shows the opposite pattern. Both lincRNAs are enriched in islets and especially in β-cells, suggesting their involvement in pancreatic development and regulation [86]. The further exploration of lincRNAs in pancreatic developmental regulation is necessary to fully understand their roles.

## 14. Conclusions

The study of lincRNAs has significantly expanded our understanding of gene regulation during fetal development. The current review provides a comprehensive list of lincRNAs involved in fetal development (Table 1). Traditionally, the focus of gene expression research was centered on protein-coding genes, but emerging evidence highlights the indispensable roles of lincRNAs in shaping developmental processes. These molecules, transcribed from intergenic regions, exhibit complex interactions with chromatin and transcriptional machinery, influencing gene expression, chromatin structure, and cellular differentiation.

Throughout various stages of fetal development, lincRNAs have been shown to play critical roles in tissue-specific regulation and organogenesis. Their involvement ranges from neural and cardiac development to reproductive system maturation and pancreas organogenesis. For instance, lincRNAs such as Fendrr and Braveheart are essential for the development of the lungs and heart, respectively, while others like Evf2 and Peril are crucial for neural differentiation and function.

Despite these advancements, the comprehensive understanding and functional annotation of lincRNAs remain incomplete. Many lincRNAs have been identified as crucial for various developmental processes, but their precise mechanisms and full range of functions are still under exploration. Future research, driven by advancements in high-throughput sequencing technologies and functional genomics, promises to reveal further insights into the regulatory networks governed by lincRNAs. Such discoveries could pave the way for novel diagnostic and therapeutic strategies, enhancing our ability to address developmental disorders and diseases linked to dysregulated noncoding RNAs.

Overall, the study of lincRNAs is reshaping our perspective on gene regulation, underscoring the importance of these previously overlooked molecules in fetal development and their potential impact on future biomedical research.

## Figures and Tables

**Table 1:** List of lincRNAs known to be involved in fetal development.

	Strain	Observed Effect	References
1	Star-Nat	Reproductive system development (expressed in ovarian tissues)	[81,82]
2	NORFA	Reproductive system development (fertility regulation in the ovary)	[81,82]
3	TCONS_00000891	Cardiac development	[77]
4	TCONS_00028686	Cardiac development (expressed in embryonic and adult heart)	[77]
5	TCONS_00009015	Cardiac development	[77]
6	βlinc1	Immune system development (β-cell regulation)	[85]
7	βlinc2	Pancreatic development	[86]
8	βlinc3	Pancreatic development, particularly in βlinc2-cell regulation.	[86]
9	Braveheart (Bvht)	Cardiac development	[73,75]
10	Fendrr	Organogenesis (lung, heart, and GIT)	[44,67]
11	linc-Brn1a	No observed effect	[44]
12	linc-Brn1b	CNS development	[44,69]
13	Peril	Organogenesis (lung, heart, and GIT)	[44]
14	Tug1	No observed effect	[44]
15	linc-Cox2	No observed effect	[44]
16	Fabl	No observed effect	[44]
17	linc-Enc1	No observed effect	[44]
18	Manr	No observed effect	[44]
19	Haunt	No observed effect	[44]
20	Hotlip	No observed effect	[44]
21	Mdgt	Organogenesis (lung, heart, and GIT)	[44]
22	Celr	No observed effect	[44]
23	Cmde	No observed effect	[44]
24	Spasm	No observed effect	[44]
25	linc-Pint	No observed effect	[44]
26	linc-p21	No observed effect	[44]
27	linc-Ppara	No observed effect	[44]
28	Evf2 (Dlx6os1)	CNS development (interneuron development in the brain)	[69]
29	lincRNA **H19**	Organogenesis (lung, heart, and GIT)	[83,84]
